# Editorial: AI innovations in neurological and psychiatric disorder management: diagnosis to treatment

**DOI:** 10.3389/fnhum.2025.1759844

**Published:** 2026-01-05

**Authors:** Jin Hong, Yang Bai, Yuxiu Sui, Junming Jian

**Affiliations:** 1 School of Information Engineering, Nanchang University, Nanchang, China; 2 Affiliated Rehabilitation Hospital, Jiangxi Medical College, Nanchang University, Nanchang, China; 3 Nanjing Brain Hospital Affiliated to Nanjing Medical University, Nanjing, China; 4 Jiangxi Cancer Hospital, Nanchang, China

**Keywords:** artificial intelligence, diagnosis and treatment, disorder management, neurological disorder, psychiatric disorder

## Introduction

Neurological and psychiatric disorders affect over 1 billion people globally, posing immense health, economic, and social burdens. From neurodevelopmental conditions (ADHD, dyslexia) to cerebrovascular events (stroke), age-related cognitive impairment (MCI), and traumatic brain injury (TBI), these disorders share core challenges: complex pathogenesis, heterogeneous presentations, delayed diagnosis, and limited personalized care. Traditional subjective assessments and one-size-fits-all interventions often yield suboptimal outcomes, creating an urgent need for innovative solutions.

Artificial intelligence (AI)—encompassing machine learning, deep learning, and data analytics—has emerged as a transformative tool, harnessing multi-modal data (neuroimaging, clinical metrics, behavioral assessments) to enable precise pattern recognition, predictive modeling, and adaptive interventions. This Research Topic gathers five cutting-edge studies translating AI advancements into clinical practice across diverse brain disorders. Below, we synthesize their core contributions, contextualizing their significance in advancing AI-driven brain health.

## Summary of contributing articles

*Artificial intelligence in ADHD: a global perspective on research hotspots, trends and clinical applications* (Wang et al.) provides a comprehensive bibliometric analysis of global AI research in ADHD, a neurodevelopmental disorder affecting 5–7% of children and adolescents. The study maps key research hotspots over the past decade, including AI-driven diagnostic tools (e.g., based on EEG and behavioral data), personalized intervention design (such as adaptive digital therapies), and neuroimaging-based subtype classification to tailor treatment. It identifies critical gaps between research and clinical practice—including limited multi-center validation, inconsistent data standards, and accessibility barriers in low-resource regions—and offers actionable recommendations to guide future AI development for ADHD, emphasizing the need for scalable, age-appropriate, and equitable solutions.

*Use of artificial intelligence in the management of stroke: scoping review* (Sierra et al.) systematically synthesizes AI applications across the entire stroke care pathway, from prevention and acute triage to rehabilitation and long-term outcome monitoring. The review highlights AI's role in accelerating acute stroke diagnosis (e.g., automated CT/MRI lesion detection to reduce door-to-treatment time), predicting stroke risk and functional recovery trajectories, and optimizing personalized rehabilitation plans (such as adaptive physical therapy algorithms). It underscores how AI streamlines clinical workflows, reduces human error, and addresses disparities in stroke care, while noting persistent challenges in data standardization, algorithm interpretability, and integration into existing healthcare systems—aligning with the Topic's focus on translating AI innovation into real-world clinical impact.

*Reaction time variations in normal aging and elderly MCI patients under various cognitive load conditions* (Zhu et al.) fills a critical gap in the early detection of MCI, a precursor to Alzheimer's disease and other dementias. The study recruited 216 elderly participants (108 with MCI and 108 healthy controls) to measure reaction times across graded cognitive load tasks (simple attention, working memory, and executive function). By identifying distinct response patterns—including slower reaction times and increased variability in MCI patients, particularly under high cognitive load—the research provides a foundational dataset for developing AI-driven predictive models. These non-invasive, low-cost metrics, when integrated with neuroimaging or genetic data, hold promise for population-wide MCI screening, enabling early intervention to delay cognitive decline.

*Advancements in the application of multimodal monitoring and machine learning for the development of personalized therapeutic strategies in traumatic brain injury* (Wei et al.) focuses on TBI, a leading cause of death and disability among young adults and the elderly. The study demonstrates how machine learning algorithms integrate real-time multimodal data—including EEG for neural activity, intracranial pressure sensors, hemodynamic metrics, and clinical outcome scores—to accurately assess injury severity, predict recovery trajectories, and guide adaptive neuroprotective interventions. It addresses a key challenge in TBI care: data heterogeneity across patients, by developing robust models that account for individual differences in injury mechanism, age, and comorbidities. This work advances the Topic's core objective of personalized treatment, offering a framework for translating multimodal AI into bedside decision-making tools for TBI patients.

*Emerging technologies and neuroscience-based approaches in dyslexia: a narrative review toward integrative and personalized solutions* (Niu et al.) synthesizes AI-driven tools and neuroscience insights to address dyslexia, a common learning disorder affecting 5–17% of school-age children. The review emphasizes the value of integrating multi-modal data—including fMRI-derived brain connectivity patterns, eye-tracking metrics, and behavioral assessments—to develop personalized diagnostic frameworks and cognitive training platforms. It critiques traditional “one-size-fits-all” interventions, advocating for AI algorithms that adapt to individual learning styles and neurobiological profiles. By bridging the gap between theoretical neuroscience and clinical practice, the study provides a roadmap for developing accessible, evidence-based AI tools to support dyslexic learners worldwide.

## Conclusion

The five studies in this Research Topic collectively showcase the breadth and depth of AI's potential to transform neurological and psychiatric care. From mapping global research trends in ADHD and optimizing stroke care pathways, to enabling early MCI screening, personalizing TBI management, and advancing dyslexia interventions, each contribution aligns with the Topic's core mission: leveraging data-driven innovation to address unmet clinical needs. Together, they highlight three key themes: the power of multi-modal data integration, the importance of personalized care, and the need to bridge research-practice gaps for equitable AI translation.

